# Nanodiamond-Enhanced Nanofiber Separators for High-Energy Lithium-Ion Batteries

**DOI:** 10.1021/acsami.3c04305

**Published:** 2023-06-26

**Authors:** Aashray Narla, Wenbin Fu, Alp Kulaksizoglu, Atsushi Kume, Billy R. Johnson, Ashwin Sankara Raman, Fujia Wang, Alexandre Magasinski, Doyoub Kim, Mohammed Kousa, Yiran Xiao, Samik Jhulki, Kostiantyn Turcheniuk, Gleb Yushin

**Affiliations:** † School of Materials Science and Engineering, Georgia Institute of Technology, Atlanta, Georgia 30332, United States; ‡ Sila Nanotechnologies Inc., Alameda, California 94501, United States; § Daicel Corporation, 1239, Shinzaike, Aboshi-ku, Himeji, Hyogo 671-1283, Japan

**Keywords:** nanodiamonds, electrospinning, nanofibers, separators, Li-ion batteries

## Abstract

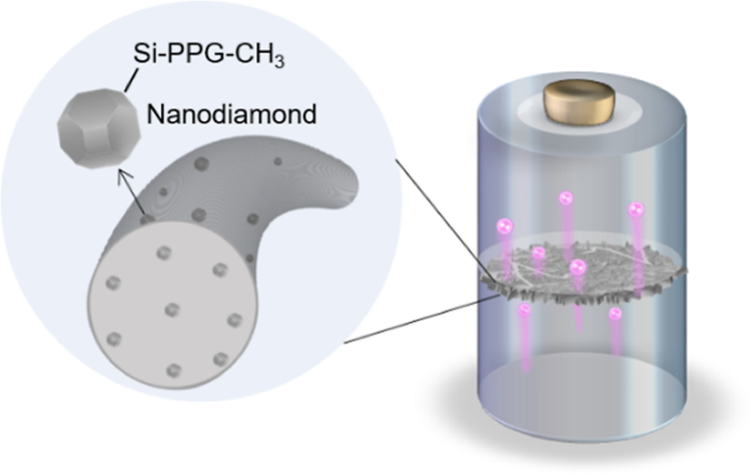

Current lithium-ion battery separators made from polyolefins such as polypropylene and polyethylene generally suffer from low porosity, low wettability, and slow ionic conductivity and tend to perform poorly against heat-triggering reactions that may cause potentially catastrophic issues, such as fire. To overcome these limitations, here we report that a porous composite membrane consisting of poly(vinylidene fluoride-*co*-hexafluoropropylene) nanofibers functionalized with nanodiamonds (NDs) can realize a thermally resistant, mechanically robust, and ionically conductive separator. We critically reveal the role of NDs in the polymer matrix of the membrane to improve the thermal, mechanical, crystalline, and electrochemical properties of the composites. Taking advantages of these characteristics, the ND-functionalized nanofiber separator enables high-capacity and stable cycling of lithium cells with LiNi_0.8_Mn_0.1_Co_0.1_O_2_ (NMC811) as the cathode, much superior to those using conventional polyolefin separators in otherwise identical cells.

## 1 Introduction

With climate change being an imminent concern, there is a growing need for environmentally friendly renewable energy sources (such as solar and wind) as well as efficient energy storage devices.^1^ Lithium (Li)-ion batteries (LIBs) are ideal energy storage devices due to their high energy, power density, efficiency, long cycle life, and low self-discharge.^2^ The demand for LIBs is further fueled by the automotive industry and the growing usage of portable smart devices.^3^ Over the past decade, extensive research has shown pathways for improving energy density of LIBs by incorporating high-voltage cathodes, conversion-type electrodes, solid-state electrolytes, silicon, or Li metal anodes (as in Li-metal batteries, LMBs).^4−7^ Despite the advances in LIBs/LMBs with high energy density, many safety concerns still remain, which could hinder their wide scale adoption. Separators, an inactive component in batteries, do not directly participate in redox events but physically separate battery anode and cathode, prevent short circuits, and serve as reservoirs of liquid electrolytes and as conduits for Li-ion transport between the electrodes.^8^ In general, separators play a pronounced role in determining battery rate capability, lifespan, and perhaps most importantly, safety.^9−11^

Conventional separators are made from thermoplastic polyolefins such as polyethylene (PE) and polypropylene (PP), and they can be produced at a large scale but they tend to suffer from low porosity, poor thermal stability (excessive shrinking at elevated temperatures that may induce internal shorts), and high tortuosity, which are likely to cause premature failures of cells having high energy density.^12,13^ To illustrate, as LIBs are cycles for extended periods of time, heat-triggered exothermic “thermal runaway” reactions occur due to large overpotentials present in the cells.^3,14^ These reactions lead to an overall increase of temperature in the cell, which could cause the separator to shrink, the solid electrolyte interphase (SEI) to break down, and eventually short-circuit, resulting in explosions and fire in some (fortunately rare) cases, a relatively common shortcoming of PE and PP separators. A separator that minimizes the overpotentials in the first place through a facile conduction of Li ions, and that is aided with tools (i.e., thermally stable and able to prevent shrinkage at high temperatures) to dissipate heat in the case of a thermal runaway, is required to reduce the probability of short circuiting, improve the cycle life, and ensure safety.^15−17^ Furthermore, a thin, porous, and ionically conductive separator is required for the next-generation Li batteries to garner the benefits of high-energy dense electrodes.

Polymer–nanoparticle composites have been of interest for the past two decades as candidates for separators in LIBs. Nanoparticles, inorganic or organic, when embedded in the polymer matrix tend to improve mechanical, thermal, and structural properties of the composite.^18^ With widespread availability of nanostructured and nano-sized particles such as carbon nanotubes,^19^ inorganic nanowires,^20,21^ dendrimers,^22^ graphene^23^ with tunable size, and surface functionalization, there have been significant efforts to improve appropriate properties of the composite for their use as separators in high-energy-density LIBs and LMBs. Yet, shortcomings such as poor cycle life, large hysteresis, and complicated processing methods remain, which call for further innovation in their chemistry and engineering aspects.

Nanodiamonds (NDs) are a relatively unexplored class of carbon nanoparticles featuring high surface-to-volume ratio,^24^ tunable size and surface functionalization,^25^ high elastic modulus, and exceptional thermal conductivity.^26^ They have been explored toward various applications such as in biomedical fields,^27^ supercapacitors,^28,45^ and batteries.^29−31^ Spraying NDs on a commercial separator can provide favorable electrolyte affinity for regulating Li^+^ distribution, high Young’s modulus against Li dendrite growth, and excellent thermal diffusion ability.^29^ Recent studies also revealed that NDs can be used as an additive to liquid electrolytes to suppress the growth of lithium dendrites,^30^ and ND thin films can provide interfacial protection for Li metal anode.^32^ Considering these unique properties of NDs, in this study we developed a functionalized composite separator based on NDs incorporated with poly(vinylidene fluoride-*co*-hexafluoropropylene) (PVDF-HFP) nanofibers and characterized their thermal, mechanical, and morphological properties, which all deemed suitable for use in high-energy dense LIBs. Then, we explored the electrochemical properties of these separators in LIBs using high-voltage LiNi_0.8_Mn_0.1_Co_0.1_O_2_ (NMC811) as the cathode. We report excellent performance of these separators in these cells, especially when compared to standard PP separators. This study reintroduces NDs for composite membrane fabrications, opening up a wide variety of possibilities for their future use.

## 2 Experimental Section

### 2.1 Preparation of ND-Functionalized Membranes

ND dispersions (1 wt %) were prepared by mixing 0.017 g of ND-Si-PPG (Daicel Corporation), 2.41 g of *N*,*N*-dimethylacetamide (DMAc, Sigma-Aldrich, purity ≥99.5%), and 6.52 g of acetone (Sigma-Aldrich, purity ≥99.8%). The 5 wt % ND dispersions were prepared by mixing 0.11 g of ND-Si-PPG, 2.41 g of DMAc, and 6.52 g of acetone. The dispersions were stirred for 30 min at a speed of 300 rpm, and to each solution was added 1.64 g of PVDF-HFP (Sigma-Aldrich avg. *M*_w_ ∼ 455,000). The subsequent mixtures were stirred at 50 °C for 24 h to achieve a homogeneous and viscous gel. The mixed gel was tip-sonicated (Misonix S-4000 at 1 W) for 1 min before electrospinning to further improve the dispersion. Electrospinning was conducted using 3 mL of dispersion for each sample in a syringe and then secured onto a syringe pump to extrude the slurry at a rate of 0.5 mL/h with a rotating drum at 150 rpm. The distance and accelerating voltage between the syringe’s tip and the rotating drum were 16 cm and 16 kV. After electrospinning, the subsequent membrane was separated from the aluminum sheet, folded in half, and hot pressed at 110 °C for 2 h under a pressure of 30 MPa using press (Across International, Swingpress) to decrease the porosity. The membranes were then dried for 24 h at 80 °C for subsequent studies.

### 2.2 Material Characterizations

A Hitachi SU8230 scanning electron microscope (SEM) was used to take images of the membrane structure after electrospinning. Single polymer composite fibers and ND plane distance were determined by using a transmission electron microscope (TEM, Tecnai G2 F30). Thermogravimetric analysis (TGA) was carried out using TA instruments TGA (TA Q600), where the heat was ramped up from room temperature to 400 °C at 5 °C/min. Differential scanning calorimetry (DSC, TA instruments Q200) was used to quantify the % crystallinity of the samples as a function of the ND concentration. The temperature of the samples was ramped from room temperature to 160 °C and cooled back to room temperature at 10 °C/min. The test was run four times to ensure consistent results between cycles. X-ray diffraction (XRD) was performed on a Panalytical XPert PRO Alpha-1 XRD instrument by placing the samples on a zero-background holder. The chemical bonding features of produced samples were studied using Fourier transform infrared spectroscopy (FT-IR) on Thermo Scientific Nicolet 6700 (USA).

To characterize the stress–strain behavior of the separators, tensile tests were performed following ASTM D882 Standards using a 25 N force gauge on a Mark-10 ESM303 test stand. Gurley values were obtained by measuring the time taken for 100 mL of air to pass through a fixed area (19.6 cm^2^) under a pressure of 0.02 MPa using a Gurley Precision Instrument (TROY). The porosity of separators was evaluated by the absorption experiment of *n*-butanol for 10 h, as reported by previous studies and calculated based on the eq 1.^21,33^
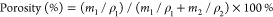
1where *m*_1_ and *m*_2_ are the weights of pristine and *n*-butanol-saturated separators and ρ_1_ and ρ_2_ are the densities of the separator polymer and *n*-butanol, respectively. The surface wettability of separators was investigated by static contact angle measurements on a drop shape model 250 goniometer (USA) with a drop of electrolyte solution onto the surface of separator membranes. The electrolyte solution is 1 M LiPF_6_ dissolved in ethylene carbonate (EC) and diethyl carbonate (DEC) (50:50 v/v %). To calculate the electrolyte uptake, the separators were immured in the electrolyte solution for 2 h, and the weight was measured before (*M*_0_) and after (*M*_1_) electrolyte absorption based on eq 2
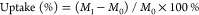
2

The sample preparation for the pulsed-field gradient (PFG) NMR experiment is very similar to that reported previously.^34^ Polymer composites were thoroughly dried at 80 °C for 24 h in a vacuum oven. Then, the polymer composites were stacked together into a 4 mm disc and loaded into a 5 mm diameter NMR probe. The 1 M LiPF_6_-EC/DEC solution was dropped into the NMR tube to completely soak the membranes. A Bruker AVIIIHD-500 NMR instrument was used to measure diffusion coefficients for different nuclei (*D*_Li_, *D*_H_, and *D*_F_) at 25 and 40 °C. The PFG spin-echo NMR technique was used to probe the nuclear species: ^7^Li at 116.8 MHz, ^19^F at 282.7 MHz, and ^1^H at 300.5 MHz.

### 2.3 Cell Assembly and Electrochemical Tests

In an argon environment inside a glovebox (H_2_0 < 0.1 ppm), 2032-type coin cells were assembled using a commercial NMC811 electrode (NEI Corporation, mass loading, ∼10 mg/cm^2^) as the cathode, dried membrane as the separator, Li metal as the reference/counter electrode, and 70 μL of 1 M LiPF_6_ in EC/DEC (1:1, v/v %) as the electrolyte. Cyclic voltammetry (CV) was performed using a Gamry Potentiostat at a cycling rate 0.1 mV s^–1^ with a step size of 1 mV. The electrochemical stability of separators was evaluated by using liner sweep voltammetry (LSV) for a coin cell of Li/separator/stainless steel from 3 to 6.5 V (vs Li/Li^+^) at a scan rate of 10 mV s^–1^ on a Gamry Potentiostat. The cycling stabilities of the membranes were tested at different C-rates (1C = 190 mA h g^–1^) using an Arbin system. To calculate the ionic conductivity, electrochemical impedance spectroscopy (EIS) was performed in the frequency range of 1 MHz–0.1 Hz at room temperature.

## 3 Results and Discussion

**Scheme 1:**
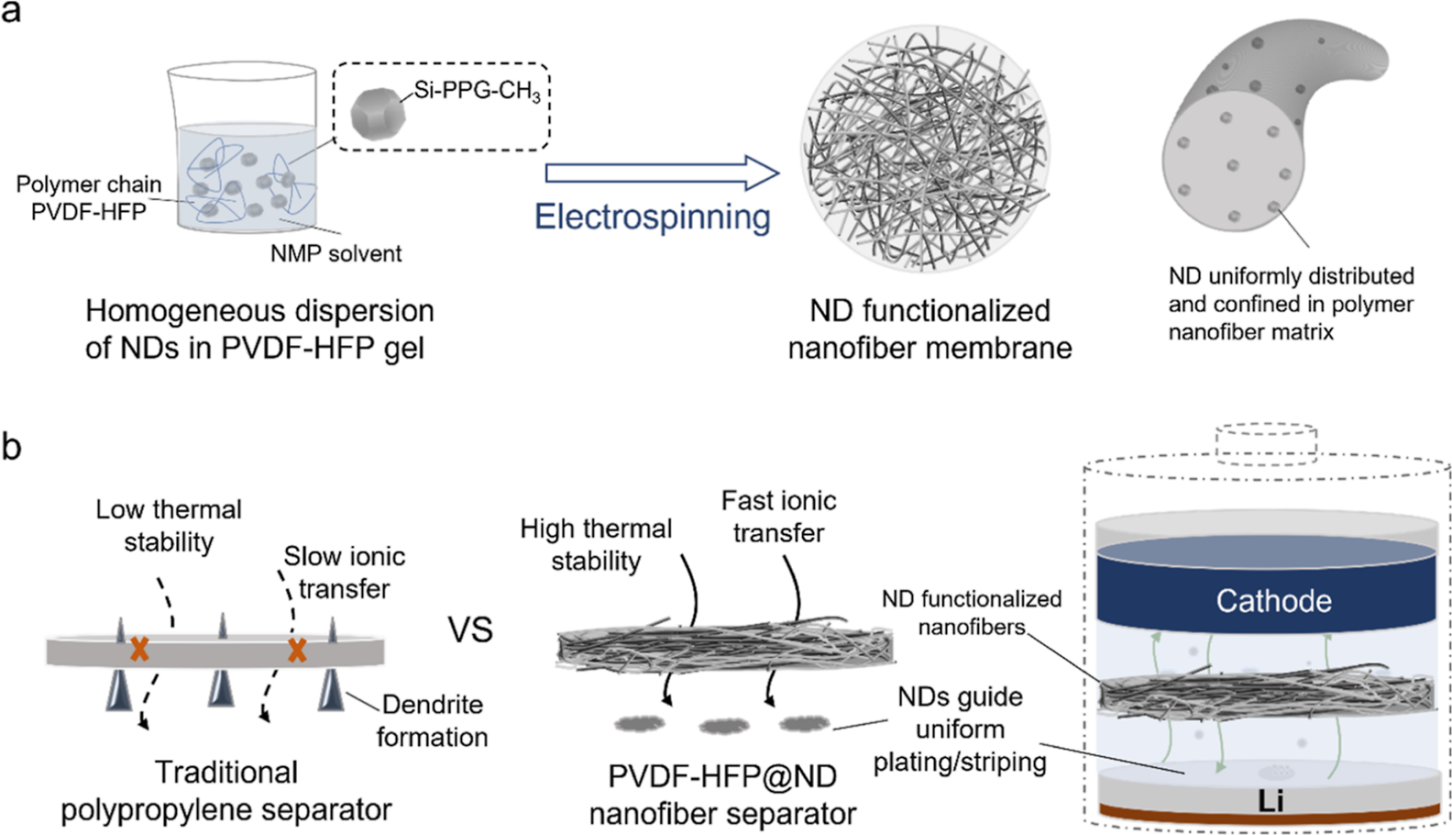
(a) Schematic of the Synthesis of ND-Functionalized Nanofiber Separator and (b) Its Advantages over Traditional Polypropylene Separator in Li Batteries

In this study, ND-functionalized nanofiber membranes can be produced by electrospinning, as illustrated in Scheme 1a. The NDs were produced by Daicel Corp. and surface-functionalized using ball milling of polypropylene glycol (PPG) and silane coupling agent (ND-Si-PPG-CH_3_) and then dispersed in DMAc to form a homogeneous dispersion (details are given in the Experimental Section). To prepare the polymer gel for electrospinning, PVDF-HFP and NDs in a mixed solvent were prepared by dissolving PVDF-HFP into a mixture of DMAc and acetone (Figure S1, Supporting Information). In this process, we conducted tip ultrasonication to improve the homogeneity of ND distribution in the polymer matrix. The resulting ND/PVDF-HFP/NMP mixed gel was electrospun onto an aluminum foil at a high voltage (16 kV) to form a free-standing, non-woven membrane of PVDF-HFP nanofibers intercalated with NDs. The membrane was then dried and hot pressed for future tests and use. For comparison, we produced membranes with ND mass fractions of 0, 1, and 5% (ND by weight relative to the polymer), which were denoted as PVDF-HFP, PVDF-HFP@1%ND, and PVDF-HFP@5%ND. In general, commercial separators made from thermoplastic polyolefins such as PP or PE could suffer from low porosity, low heat resistance, and slow ionic transport, leading to low-rate capability and capacity degradation due to the sharp dendrite formation that may destroy cathode particles in the vicinity, as illustrated in Scheme 1b. In contrast to these separators, ND-functionalized nanofiber membranes could overcome these difficulties because the nanofiber network offers a higher porosity and faster ion diffusion, which could facilitate the electrolyte uptake and Li transport. NDs could guide the uniform plating/striping of Li on the Li anode surface, without the formation of sharp dendrites. Besides, the hardness and thermal stability of NDs can also improve mechanical stability and thermal stability that allow cells to operate within a wider temperature range.

**Figure 1:**
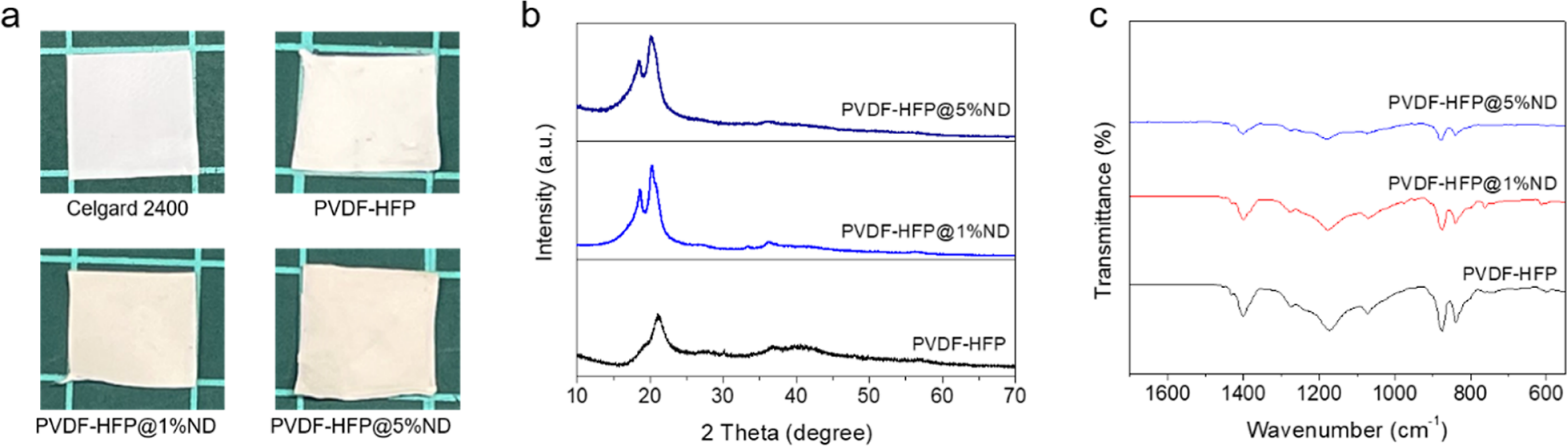
(a) Photograph of produced membranes containing 0, 1, and 5 wt % NDs. (b) XRD and (c) FT-IR profiles of the pristine polymer and composite polymers.

Figure 1a shows photographs of the produced PVDF-HFP membrane, ND-functionalized membrane, and commercial (Celgard 2400) PP separator (cut into a similar size for comparison). The uniform color and contrast indicate a homogeneous distribution of NDs within the membranes. XRD (Figure 1b) patterns of the ND-functionalized samples (both 1 and 5 wt %) are similar to that of the pure PVDF-HFP, suggesting that ND incorporation did not change the packing of polymer significantly in the solid state. However, it is noted that the employed ND concentration was too low to observe their characteristic diffraction peaks. To further identify their chemical bonding features in the membranes, we performed Fourier transform infrared spectroscopy (FT-IR, Figure 1c). In the FT-IR spectra of PVDF-HFP (0 wt % ND) and the composites with 1 and 5 wt % NDs, the bands for −CF stretching (1400 cm^–1^), anti-symmetric −CF_2_ stretching (1172 cm^–1^), CF_3_ out-of-plane stretching/bending (1072 cm^–1^), and the characteristic bands at 838, 871, and 1168 cm^–1^ due to the γ phase crystalline structure of PVDF-HFP are observed. The obtained FT-IR spectra are essentially identical to that of pure PVDF-HFP, suggesting non-destructive nature of the membrane fabrication process. After hot press, we find four stretching positions at 613, 761, 795, and 974 cm^–1^ corresponding to the α phase of PVDF (Figure S2). This could indicate a phase transition between γ to α after hot press as well as the impact from acetone as a co-solvent, as observed in the previous study.^35^

**Figure 2:**
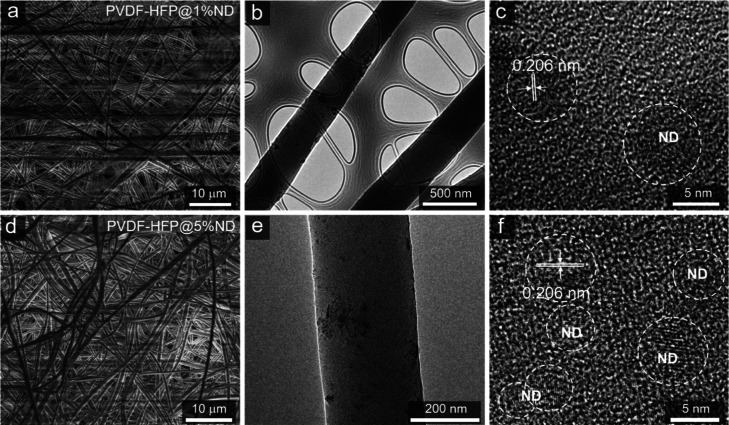
(a) SEM and (b,c) TEM images of PVDF-HFP@1%ND. (d) SEM and (e,f) TEM images of PVDF-HFP@5%ND.

Scanning electron microscopy (SEM) shows that non-woven, porous membranes composed of uniform fibers with a diameter of ca. 0.5 μm were produced (Figure 2a,d), but NDs could not be detected. To better observe the NDs and their distribution in the fibers, we performed transmission electron microscopy (TEM). The TEM images show that individual NDs are relatively uniformly dispersed in patches, and there are also domains of agglomerated NDs as well along the polymer matrix (Figure 2b,e). It is well known that both the chemical nature and ND surface (e.g., their surface functional groups) and their concentration in the initial dispersion play important roles in their agglomeration during polymer-ND composite fabrication.^36^ Our fabrication process resulted in NDs/their clusters with diameters in the range of 7–40 nm, which can be confirmed through high-resolution TEM (Figure 2c,f). The crystalline regions are present in polymer fiber represented by parallel patterns, and the distance between the parallel lines was calculated to be ∼0.204 nm which is similar to the interplanar distance of that of NDs.^37,38^ Forming a composite where NDs are not agglomerated is challenging and requires use of energy-intensive methods. Our results suggest that a homogeneous dispersion of NDs in the polymer matrix can be achieved through the surface functionalization of NDs, sonication mixing, and electrospinning process. As such, the ND dispersion enables the impartments in the thermal, mechanical, and electrochemical properties for the polymer.

**Figure 3:**
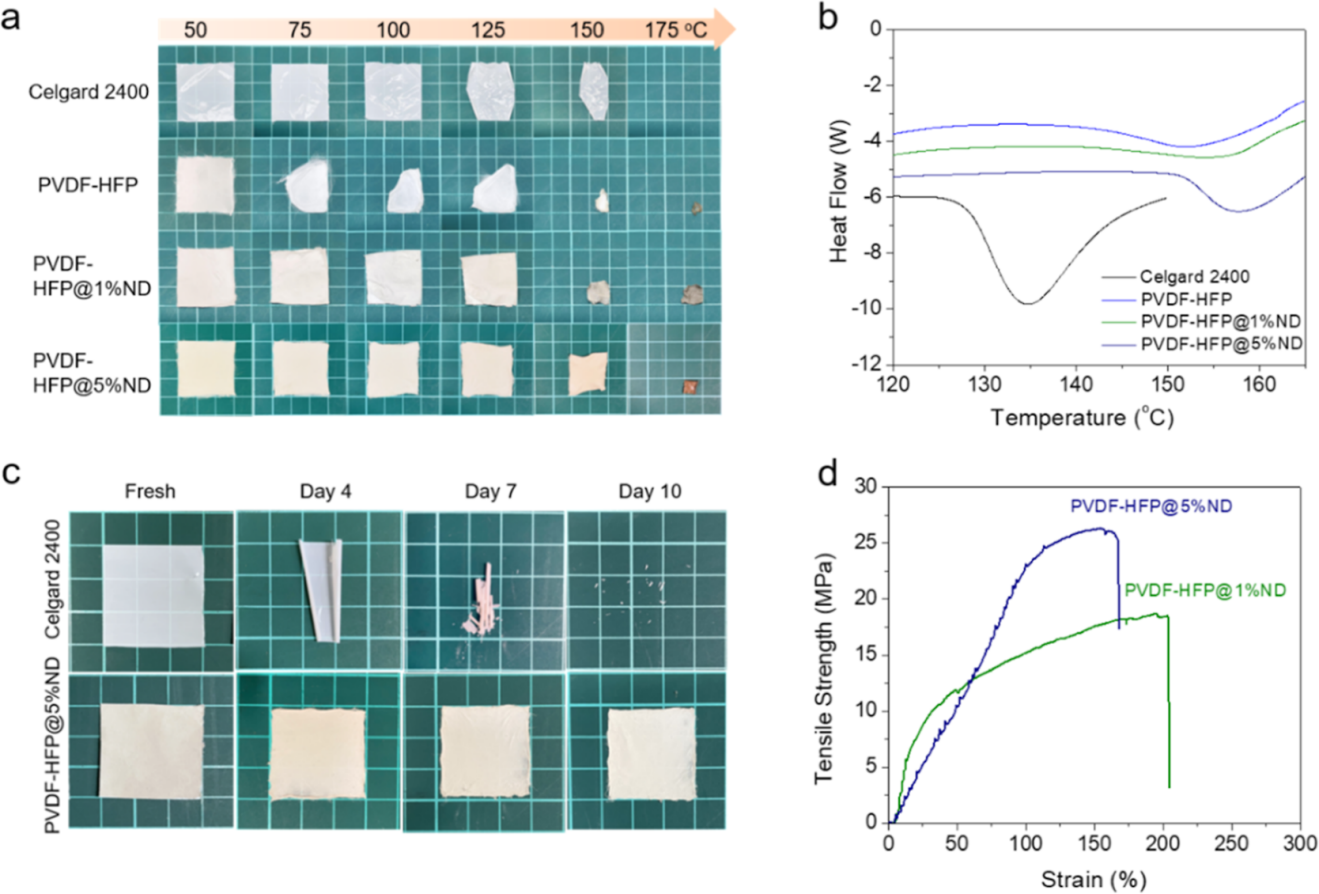
(a) Thermal stability of different membranes at temperatures from 50 to 175 °C (kept 20 min at each temperature). (b) DSC curves of different samples. (c) Shape-retention study of PVDF-HFP@5%ND and PP membranes (∼area of 9 cm^2^) at 130 °C for up to 10 days. (d) Strength–strain plots of ND-functionalized PVDF-HFP membranes after hot press.

To explore the thermal stability, we heated different membranes from 50 to 175 °C, kept them at each temperature for 20 min, and checked their thermal shrinkage. As can be seen in Figure 3a, both Celgard 2400 PP and pure PVDF-HFP exhibit rather poor dimensional stability, and their shrinkages start at as low as 125 and 75 °C, respectively. Adding NDs can significantly improve the thermal properties, with the onset of shrinkage being approximately 100 °C. It is noted that the PVDF-HFP@5%ND membrane shows much better thermal stability than PVDF-HFP@1% ND. TGA and DSC were employed to investigate the thermal properties of produced membranes. The DSC results reveal that these membrane materials are stable up to 150 °C, irrespective of the ND concentrations in the sample (Figure 3b). The decomposition temperatures (*T*_d_s) of both samples are much higher than that of a standard PP separator (130 °C), suggesting a higher heat tolerance of these membranes during processing as well as inside the electrochemical cells. DSC data reveal that increasing the ND concentration in the composite membrane results in higher melting point (*T*_m_) for the polymer (Figure 3b). Indeed, the 5 wt % ND-containing separators exhibit the highest *T*_m_ of 156 °C and are dimensionally stable at 130 °C for long periods of time (Figure 3c). This was seen by heating the polymer membrane at 130 °C for 10 days, and the area was calculated during regular intervals (Figure S3). It is noted that the membrane with 5 wt % ND has a significantly enhanced dimensional stability without a virtually shrinkage at 125 °C. In contrast, commercial PP separators cannot survive high temperatures, and they start a shrinkage at ca. 120 °C. This clearly suggests that the developed 5 wt % ND-PVDF-HFP composite membranes could serve as a better alternative to the PP separator for long-term LIB cycling as they are likely to eliminate cell failures due to short circuits caused by thermal shrinkage of the separators at elevated temperatures. Furthermore, the enhanced *T*_m_ and *T*_m_ due to ND incorporation allow flexibility in thermal processing; for example, drying of these membranes to remove volatiles can be performed at high temperatures. As the thermal and mechanical properties of PVDF-HFP@5% ND were superior compared to those made with either PVDF-HFP@1% ND or pure PVDF-HFP, the former membranes were used for further electrochemical tests.

**Figure 4:**
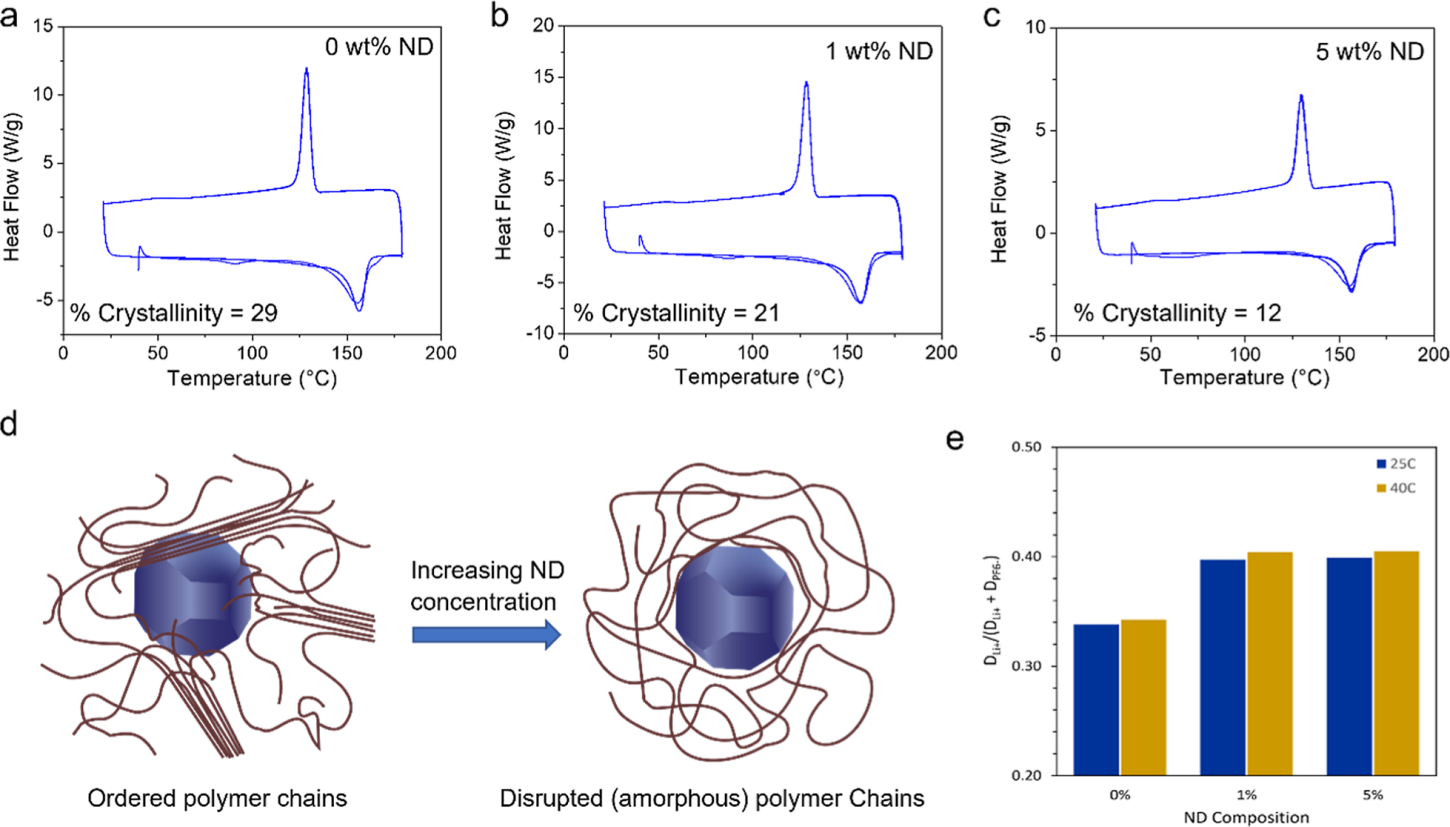
DSC cycles and crystalinity of (a) pure PVDF-HFP (0 wt % ND), (b) PVDF-HFP@1%ND, and (c) PVDF-HFP@5%ND membranes. (d) Graphical representation of how semi-crystalline polymers are disrupted by NDs to form disordered polymer chains and reduce crystallinity. (e) Li^+^ diffusion coefficients of PVDF-HFP membranes with different mass fractions of NDs calculated by NMR.

The Gurley value provides an understanding of the tortuosity and porosity of a membrane. While membranes with high tortuosity (low porosity) are desirable for high-energy dense LIBs, excessive porosity may also cause premature cell failure due to short-circuit caused by the electrodeposited Li dendrites (particularly in case of Li metal anodes) that can easily penetrate the membranes.^39,40^ Therefore, a tradeoff between porosity and tortuosity is typically necessary and is generally achieved through optimization for newly developed membranes. Our as-synthesized membranes show a very high air-permeability with an incredibly low Gurley value of only 4 s. Thus, we hot-pressed the membranes to improve the layer-to-layer stacking and reduce the air-permeability. To allow comparison of electrochemical data obtained from the use of different membranes, their Gurley values were made similar (420 s), through optimization, by densification of the membranes by hot-pressing them for different amounts of time. We observed that the PVDF-HFP@5%-ND membranes required longer time to change the porosity of membranes when compared to PVDF-HFP@1% ND under the applied pressure and heat. Based on tension tests, it is noteworthy that the tensile strength of the membranes tends to increase with the increase in the ND concentration, with the values for PVDF-HFP@5% ND and 1% ND samples being 25 and 18 MPa, respectively (Figure 4d). This difference in the mechanical strength is presumably due to a much stronger polymer–ND interaction, which can overcome the disruption in the packing of polymer chains. To further evaluate separator porosity, we conducted a *n*-butanol absorption experiment for the separators (detailed in the Experimental Section). Based on the calculations from eq 1, we find that the porosity of PVDF-HFP@5%ND is up to 71%, much higher than commercial PP separator (43%). Besides, contact angle tests (Figure S4) reveal that the ND-functionalized separator has an excellent wettability due to its small contact angle with the electrolyte. The high porosity and wettability of the functional separator ensure its higher electrolyte uptake (162%) than PP (57%).

DSC cycles were conducted in the temperature range 25–175 °C to observe the crystalline behavior change with the increase in the ND concentration (Figure 4a–c). The crystallinity (*X*_c_) of PVDF-HFP in the composites can be estimated by dividing the enthalpy of fusion (Δ*H*_f_, area under the melting curve) of the samples to that (Δ*H*_f_) of 100% crystalline pure PVDF-HFP, as shown below in eq 3.^41^
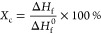
3

The crystallinity of pristine PVDF-HFP nanofibers was 29%, which reduced to 12% for 5 wt % ND-PVDF-HFP composites, clearly suggesting that the packing of the polymer chains is disrupted by the presence of NDs. NDs with their high exposed surface areas interact with the polymer chains, resulting in an overall more amorphous polymer network (Figure 4d). This engineered microstructure is beneficial for the facile conduction of Li ions across the polymer surface, which therefore would improve the Li-ion conductivity of the membranes made thereof. Furthermore, the utilized NDs have a negatively charged -Si-PPG surface functionalization,^42^ which is beneficial for improving the Li-ion conductivity via Lewis acid–base-type interactions with the Li ions. This is consistent with the data obtained from the ^7^Li NMR PFG experiment, which suggests enhanced Li-ion diffusivity (*D*_Li_) with the increase in the concentration of the NDs in the composite membranes (Figure 4e and Table S1). At 25 °C, the *D*_Li_ of 5% ND sample is 1.12 × 10^–10^ m^2^ s^–1^, which is much higher than pure PVDF-HFP (8.69 × 10^–11^ m^2^ s^–1^) and 1% ND (9.08 × 10^–11^ m^2^ s^–1^), and the Li ion transference number is also higher than that of PE-based separators, as reported in the literature.^43^

**Figure 5:**
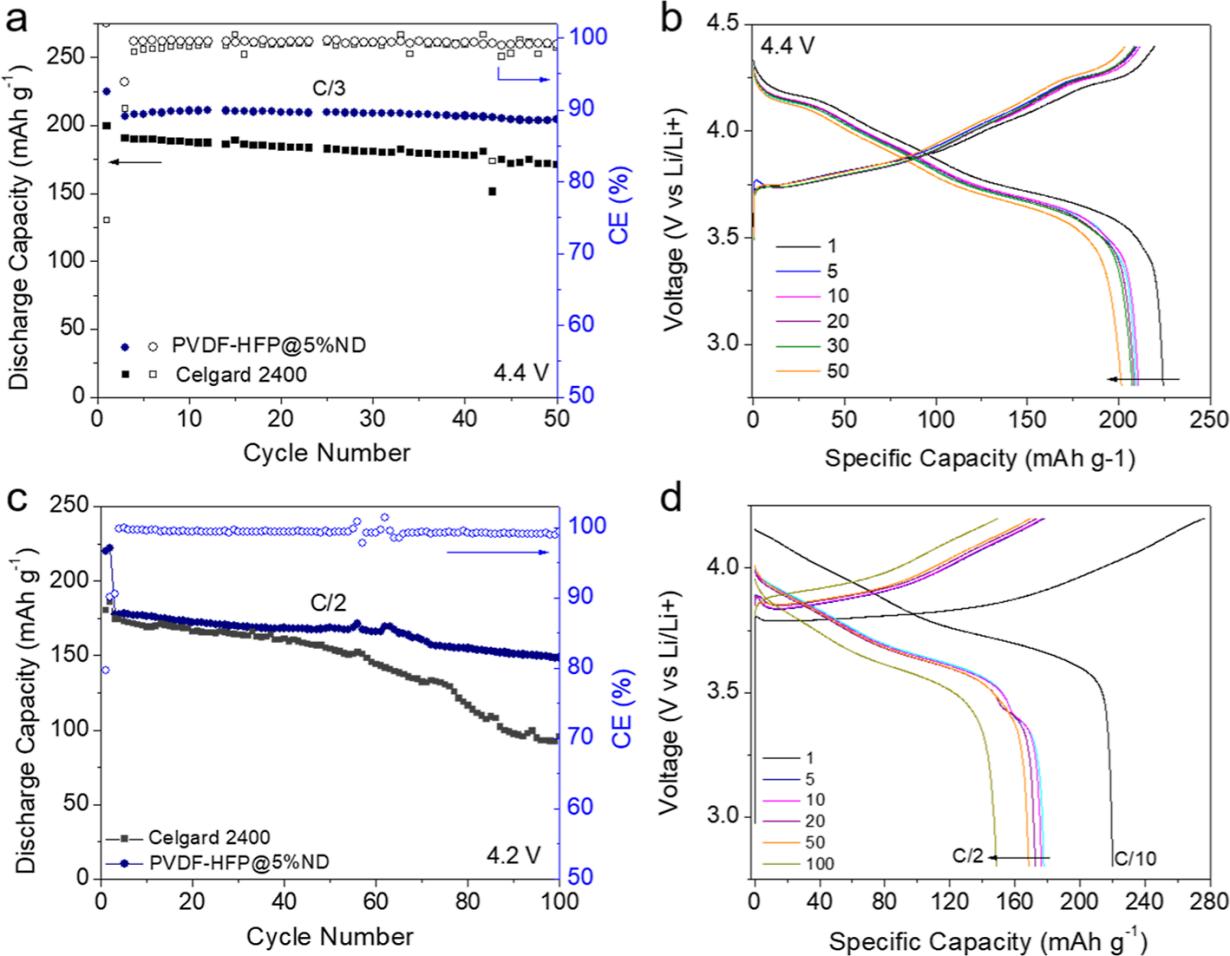
(a) Cycling performance of Li cells with Celgard 2400 and PVDF-HFP@5%ND separators in the potential range 2.8–4.4 V (vs Li/Li^+^). (b) Voltage profiles at different stages of cycling. (c) Long-term cycling performance and (d) voltage profile of the cells at C/3 between 2.8 and 4.2 V (vs Li/Li^+^).

The electrochemical properties of different separators were evaluated in coin cells (2032 type) with commercial high-loading LiNi_0.8_Mn_0.1_Co_0.1_O_2_ (NMC811) as a cathode (∼10 mg cm^–2^), Li foil as an anode (or reference electrode), and 1 M LiPF_6_ in EC/DEC as a classical electrolyte used in most publications. To check the electrochemical stability window, LSV tests were conducted for Li/separator/stainless-steel cells with different separators. From the obtained LSV curves (Figure S5), we can find that the functionalized separator (PVDF-HFP@5%ND) is more electrochemically stable than the commercial PP separator in the Li cell environment. In order to obtain high specific capacity and energy density, the assembled cells were first cycled within a voltage range of 2.8–4.4 V (vs Li/Li^+^) at a rate of C/3. As can be seen from Figure 5a, the cell with the PVDF-HFP@5%ND separator shows more stable performance and higher Coulombic efficiencies (CEs) compared to the one based on the conventional separator (Celgard 2400). The initial specific capacity is up to 210 mA h g^–1^ (stable at the 2nd cycle) and retains 204 mA h g^–1^ (∼97%) after 50 cycles, while the cell based on Celgard has a fast capacity degradation from 190 to 171 mA h g^–1^. The stable performance of ND-based separators can be confirmed by their voltage profiles at different charge/discharge cycles (Figure 5b). The plateau features are obviously stable as cycles, which means that the separator is electrochemically stable and does not cause any side reaction. Besides, the PVDF-HFP@5%ND separator can help reduce the voltage hysteresis of the NCM811 cathode (Figure S6), leading to a good reversibility with high energy efficiency. To further probe electrochemical stability of the membranes in the presence of NMC811, we cycled the cells in a lower cutoff voltage of 4.2 V (vs Li/Li^+^) at C/2, as compared in Figure 5c. The cell with ND-functionalized separator still shows better performance with a higher capacity retention of 149 mA h g^–1^ and high CE of 99.4% after cycling. The stable performance can be reflected by their voltage profiles at different cycles (Figure 5d). Besides, the CV scans at a rate of 0.2 mV s^–1^ can also suggest that this membrane is stable in the cells (Figure S7a). In addition to typical redox peaks for NCM811, there is no additional peaks that would indicate any side reactions—either due to decomposition of the separator or unwanted redox events in the presence of the separator. The electrochemical impedance was evaluated based on the Nyquist plots of the cell before and after cycling (Figure S7b**)**, the cells with the ND-functionalized separator also show a small semi-cycle after cycling, reflecting a small change in its charge-transfer resistance.

**Figure 6:**
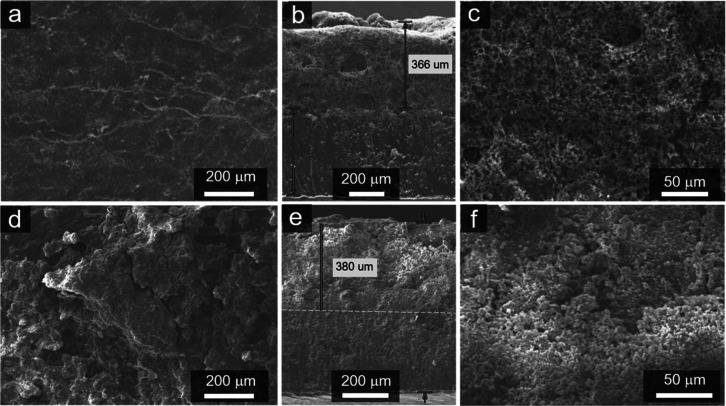
(a) Top-view and (b,c) cross-section SEM images of the Li anode from the cell with the PVDF-HFP@5%ND separator after cycling. (d) Top-view and (e,f) cross-section SEM images of the Li anode from the cell with the PP separator after cycling.

We conducted post-mortem analysis for the cells with different separators after cycling to gain a better insights into the changes in Li metal anodes and the function of separators. We captured SEM of the Li foils from different cells with ND-functionalized (PVDF-HFP@5%ND) and Celgard 2400 PP separators after cycling (2.8–4.2 V vs Li/Li^+^). From the top-view SEM images (Figure 6a,d), we can distinguish that the Li foil from the cell with the PVDF-HFP@5%ND separator is significantly smoother than the one from the cell with the PP separator that underwent non-uniform Li deposition with large dendrites. Cross-section SEM images (Figure 6b,c,e,f) show that a very thick surface layers (mixed SEI and nanostructured Li metal) with a porous structure formed on the anode surface in all cells, which is expected as Li shows poor plating stability in these electrolytes. However, with the PVDF-HFP@5%ND separator, the surface layer was very uniform and porous, whose structure could allow ions to more easily penetrate into/extract from the inner dense Li for further plating/striping processes. In contrast, with the PP separator, we noticed a non-uniform deposition/striping of Li, which could result in the poor packing layer (and slightly thickness), large dead Li particles, and cracks. These results indicate that our ND-functionalized membranes are promising as a separator in LIBs even with a Li metal as anode.

## 4 Conclusions

In summary, we demonstrated a facile electrospinning strategy for the fabrication of ND-functionalized PVDF-HFP nanofiber membranes as battery separators, and this strategy allowed us to homogeneously disperse NDs as agglomerates along the nanofiber matrix, with dominant tendency of the agglomerate formation with increasing ND concentrations. We found that even with 5 wt % ND incorporation into PVDF-HFP, which changed the polymer microstructure to be amorphous, the composites exhibited significantly improved thermal, mechanical, and electrochemical properties with respect to the pristine polymer and traditional PP separator. DSC measurements confirmed the effective role of NDs in reducing the crystalline regions (i.e., increasing local disordering polymer chains) of the polymer in the composites and improving the Li-ion conductivity. PFG experiments further confirmed that the Li^–^-ion diffusions within the membrane increase with the increase in the ND concentration. Benefiting from these promising features, the assembled cells with the ND-functionalized nanofiber separator and NMC811 cathode achieved excellent cycling stability with very small capacity loss and good rate performance, which were much better than otherwise analogous cells built by using conventional separators. This work could constitute a grand entry of NDs in the field of porous membranes and open the untapped potential of NDs for applications in LIBs and other energy storage devices.

## Supporting Information Available

Prepared PVDF-HFP/DMAc solution and PVDF-HFP/ND-PPG-CH_3_ dispersion; FT-IR of the membranes before and after hot press; degradation–time curves; diffusion coefficients for ^1^H, ^7^Li, and ^19^F; voltage hysteresis; CV curves; and Nyquist plots (PDF)
