# Supplementary material for: Nanodiamond-Enhanced Nanofiber Separators for High-Energy Lithium-Ion Batteries
Source: ACS Appl Mater Interfaces. 2023 Jun 26;15(27):32678–86. doi: 10.1021/acsami.3c04305 (PMC10347114; doi:10.1021/acsami.3c04305)
Supplement: Supplementary file 1 — am3c04305_si_001.pdf [file am3c04305_si_001.pdf]

Supporting Information

A Nanodiamond-Enhanced Nanofiber Separator for High-Energy Lithium-Ion Batteries

*Aashray Narla[1], Wenbin Fu[1,2], Alp Kulaksizoglu[1], Atsushi Kume[3], Billy R. Johnson[1], Ashwin Sankara Raman[1], Fujia Wang[1], Alexandre Magasinski[1], Doyoub Kim[1], Mohammed Kousa[1], Yiran Xiao[1], Samik Jhulki[1,2], Kostiantyn Turcheniuk[1,2], Gleb Yushin[1,2]**

[1] School of Materials Science and Engineering, Georgia Institute of Technology, Atlanta, Georgia, 30332, United States

[2] Sila Nanotechnologies Inc, Alameda, California, 94501, United States

[3] Daicel Corporation, 1239, Shinzaike, Aboshi-ku, Himeji, Hyogo, 671-1283, Japan

* Corresponding Author: yushin@gatech.edu

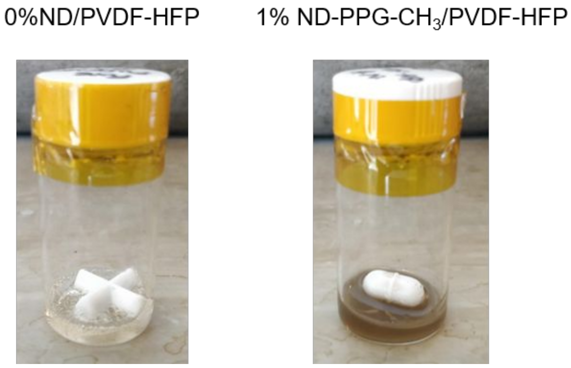

Figure S1. Photos of the prepared PVDF-HFP/DMAc solution and PVDF-HFP/ND-PPG-CH_3 dispersion.

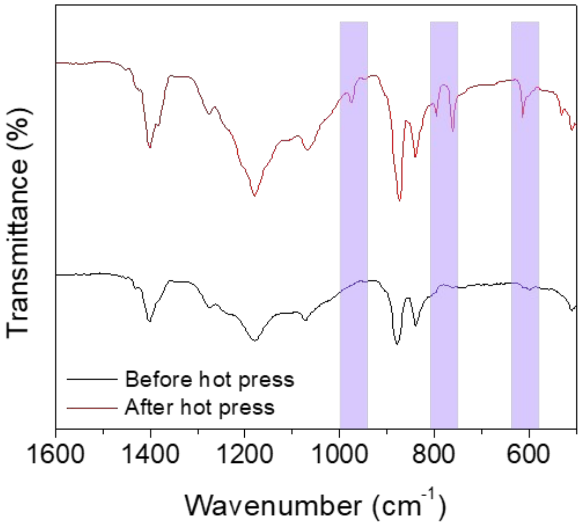

Figure S2. FTIR of the PVDF-HFP@5%ND membranes before and after hot press.

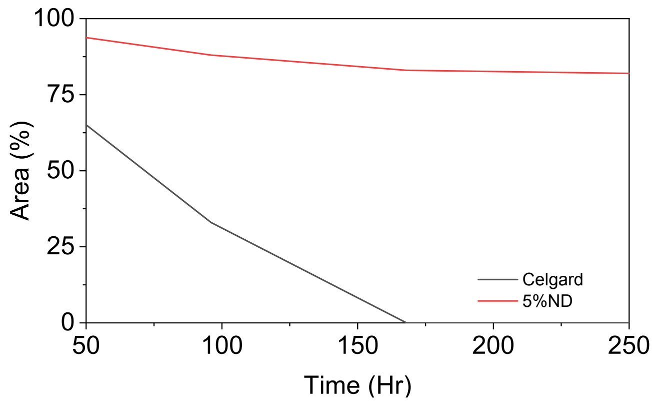

Figure S3. The degradation comparison for Celgard and ND-based membranes at 130 ℃ for 250 h. (The area was calculated during regular intervals).

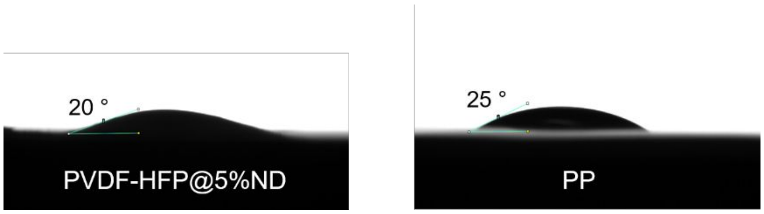

Figure S4. A comparison for the contact angles of ND functionalized and Celgard PP separators after contacting a drop of electrolyte solution for 1 s.

Table S1. Diffusion coefficients of ^{1}H, ^{7}Li and ^{19}F for samples with different ND concentrations at 25 and 40 ℃.

D [m^2 s^{-1}]		^{1}H	^{7}Li	^{19}F
Sample	Temp (°C)	EC/DEC	Li^{+}	PF_{6-}

0%	25	1.84E-10	8.69E-11	1.70E-10
	40	2.45E-10	1.07E-10	2.05E-10
1%	25	2.20E-10	9.08E-11	1.38E-10
	40	2.82E-10	1.10E-10	1.62E-10
5%	25	2.52E-10	1.12E-10	1.69E-10
	40	2.94E-10	1.53E-10	2.25E-10

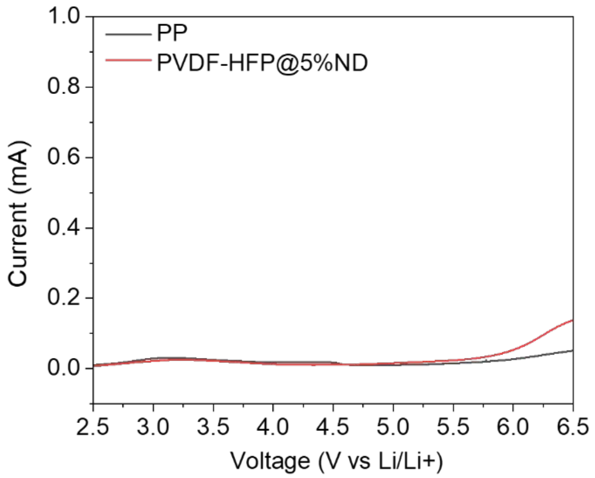

Figure S5. LSV curves of Li/separator/stainless steel cells with PP and PVDF-HFP@5%ND separators at a scan rate of 10 mV s^{-1} from 2.5 to 6.5 V (vs Li/Li+).

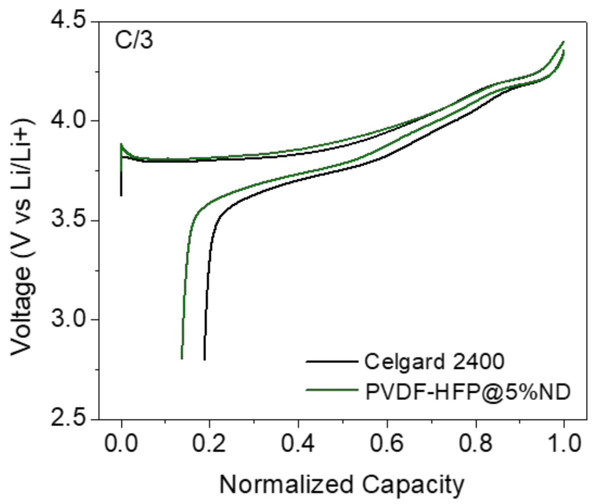

Figure S6. Voltage hysteresis of the cells using Celgard and PVDF-HFP@5%ND separators.

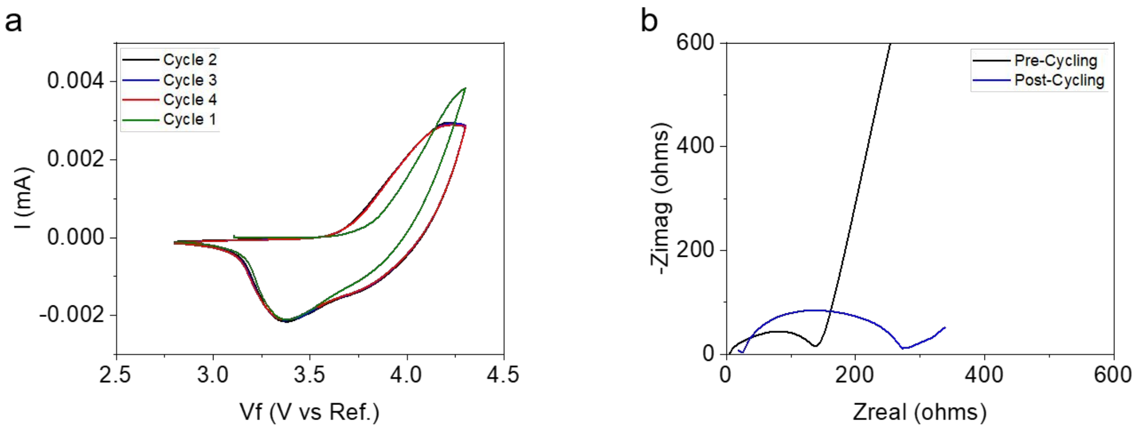

Figure S7. (a) CV curves of the cells with PVDF-HFP@5%ND separator at a scan rate of 0.2 mV s^{-1}. (b) Nyquist plots of the cells before and after long-term cycling at C/2.
